# The effect of the COVID-19 pandemic crisis on the Jordanian higher education system

**DOI:** 10.1371/journal.pone.0299531

**Published:** 2024-04-19

**Authors:** Mohammad M. Hamasha, Areen Jihad Alomari, Ala H. Bani-Irshid

**Affiliations:** Department of Industrial Engineering, Program of Desaster and Crises Management, Faculty of Engineering, The Hashemite University, Zarqa, Jordan; Al-Ahliyya Amman University, JORDAN

## Abstract

This study investigates the impact of COVID-19 pandemic-induced E-learning in Jordanian higher education. Through a quantitative survey, the study analyzes the independent variables of system use and user satisfaction, finding that information quality and service quality significantly affect these variables and that user satisfaction notably impacts E-learning. System usage moderates these effects. This research comprehensively analyzes the effects of the COVID-19 epidemic on Jordanian higher education, focusing on E-learning. It shows how information, system, and service quality affect system use and user satisfaction. The study also emphasizes these aspects’ importance in E-learning platform effectiveness. The study offers actionable insights and recommendations to help Jordan establish more resilient and effective educational policies and practices that can adjust to higher education shocks. The study recommends establishing a specialized department to modify student intention to use E-learning systems, not only during the pandemic crisis but also after-ward, to improve familiarity with E-learning tools. This study provides insights into the pandemic’s impact on Jordan’s higher education system and suggests future approaches to enhance E-learning platforms. It contributes to the development of effective E-learning systems that can improve higher education standards by pinpointing the key effects of the pandemic on the independent variables and offering workable solutions. The study emphasizes the importance of information and service quality in improving user satisfaction and system usage in E-learning.

## 1. Introduction

The COVID-19 pandemic crisis disrupted life globally in 2020, with the resulting virus spreading worldwide and impacting the economies of several countries [1]. As in any other sector, the pandemic also had a significant influence on education. In response to the unpredictable and uncontrolled nature of the pandemic, governments worldwide implemented steps to minimize its spread, including suspending face-to-face education and assessments. Most higher education systems, including Jordan’s, decided to transition to E-learning systems due to the way in which social distancing had become a valued solution to help stop the spread of the virus.

Just like in other nations, the pandemic presented Jordan’s university system with its own set of problems. The abrupt transition to online education, prompted by policies meant to discourage interaction between students, marked a significant break from conventional methods of instruction. To keep educational programs running while protecting students and employees, there was an urgent need to switch to digital learning systems. A number of social, economic, and pedagogical issues were also brought to light.

Higher education institutions in Jordan had been slowly but steadily embracing online courses before the outbreak. Because of the epidemic, there was an immediate need to move to online platforms, which tested the current digital infrastructure and exposed its strengths and shortcomings. Institutional readiness to execute successful online learning tactics varied, and there were already significant gaps in students’ and teachers’ digital access and knowledge.

Examining the quick transition to E-learning as a response to the COVID-19 pandemic, this paper explores the evolution of Jordan’s higher education system. By examining the relationship between information and service quality, it hopes to deduce how this change has affected system use and user happiness. Beyond only looking at these shifts, the report delves into the bigger picture of how Jordan’s higher education system will adapt to an increasingly digital world. Also, this study hopes to add to the global conversation about the pros and cons of E-learning, which is important because the COVID-19 pandemic is huge and has affected education systems all over the world. Examining the Jordanian setting, the study provides insights that could be applicable to other nations dealing with comparable issues in adjusting their university institutions to the post-pandemic reality. By emphasizing the importance of high-quality systems and services in ensuring user happiness and considering the possible long-term effects of this change, this enlarged introduction lays the groundwork for an in-depth examination of the adoption of E-learning in Jordanian higher education. This research intends to help higher education systems in Jordan and around the world become better prepared to withstand and recover from future disasters.

## 2. Literature review

The COVID-19 pandemic has had significant and multidimensional effects on education worldwide. A shift from traditional classroom teaching to online learning posed severe challenges to students, educators, and education systems [2, 3]. Bozkurt and Sharma [4] have suggested that the digital divide, as well as the lack of digital literacy among teachers and students, aggravated these challenges. The issue of mental health has been another key concern, with research indicating an increase in anxiety and stress levels among students [5, 6]. In contrast, evidence suggests that online learning platforms can enhance the learning process, given the adequate infrastructural and capacity supports [7, 8]. The implications on students’ performance have been varied. While Bao [9] identified a decline in academic performance, Ali [10] noted potential improvements in self-directed learning. Finally, Reuge et al. [11] indicated significant inequalities in access to education, disproportionately affecting children from low-income families and developing countries.

Fortunately, contemporary technological tools have been deployed to mitigate the challenges of distance education imposed by the pandemic. Numerous countries have resorted to E-learning channels, national television, educational platforms, learning portals, video conferencing, YouTube, mobile applications, and online classes to bolster the efficacy of distance education. Nonetheless, this abrupt transition has impacted the educational process for both teachers and students, posing significant challenges for universities and E-learning portal systems in their ability to maintain engagement between teachers and students. The COVID-19 lockdown significantly amplified the challenges of distance learning. Not all students had equal access to required technology and stable internet connectivity, thereby widening the existing digital divide [2]. Many students, parents, and teachers faced difficulties due to limited digital literacy and unfamiliarity with online teaching tools. The absence of face-to-face interaction led to reduced social engagement and isolation. Evaluating students’ performances with online assessments also became a complex issue, raising concerns about academic integrity. The sudden shift to distance learning illuminated educational inequities, with disadvantaged students facing greater hurdles due to lack of resources and support. Moreover, for courses requiring practical, hands-on experience, the transition to an online format posed significant challenges [4].

In a study conducted by Draissi, Z and ZhanYong [12], the authors sought to elucidate the response plan to the COVID-19 outbreak and the execution of distance education in Moroccan universities. The researchers adopted an applied content analysis methodology grounded in media analysis and in the assessment of diverse printed materials and documented contents, including news articles, daily newspapers, reports from the Ministry of Education, governmental publications, and notifications from universities’ websites. The study primarily employed media analysis as it has a significant role in interpreting and representing ideas about public policy, thereby sketching a comprehensive picture of distance education as a response to the COVID-19 outbreak.

The study highlighted the COVID-19 pandemic as a daunting crisis for universities, underscoring the need for continued effort to mitigate these difficulties by encouraging students and professors to invest in scientific research. Concurrently, the researchers noticed that the new teaching methodologies during the pandemic were predicated on fostering greater independence for students and ensuring effective engagement of professors by maintaining their work momentum remotely, providing free access to select paid E-learning platforms or databases. Yulia [13] spearheaded a descriptive study to identify the ways the coronavirus pandemic influenced the restructuring of education in Indonesia. The study elucidated the strategies and processes of online learning that educators worldwide employed during university closures to limit the spread of the virus. It validated the significance of diverse strategies to enhance the efficacy and fluidity of online education and evidenced the positive impact of internet-based learning. The study also observed a considerable impact of the pandemic on the education system, particularly after the substitution of traditional learning methods with online education.

Hasballah [14] aimed to spotlight the challenges faced by Islamic higher education institutions during the COVID-19 pandemic, specifically in Indonesia. The research pinpointed the maintenance of the quality of teaching as the critical challenge, a difficulty largely attributed to financial constraints. Moreover, the study mentioned other challenges, such as the need for families to prepare students to adapt to new strategies of online education and the obligation for educators to modify their teaching methods to suit the pandemic’s demands. Saleh and Abu Al-Qasim [15] conducted a study to assess the extent of student adaptation and engagement post-transition to distance learning during university closures caused by the pandemic. Using a descriptive analytical method and various statistical tools, the study analyzed pedagogical skills based on a sample of 100 students from the Faculty of Economics at Ghardaia University. Their results underscore a general willingness to adopt distance learning, yet also reveal the barriers limiting student engagement with activities on different platforms. Zhou’s [16] research discussed the impact of independent variables on user satisfaction, suggesting that service quality was a primary determinant of trust, and that system quality significantly influenced satisfaction. Additionally, Yan and Yang [17] showed that user trust was significantly swayed by perceived ease of use.

Abdelfattah et al. [18] examines the major obstacles that affected the use of online learning platforms in universities and colleges during the epidemic. There is a strong correspondence between this and the paper’s investigation of how COVID-19 affected Jordanian universities and online education. The study and [18] both stress the importance of behavioral, organizational, and technological aspects in how the pandemic affected e-learning. The challenges to successful E-learning adoption during times of crisis can be better understood with the help of this synergy. The paper’s analysis of the impact of the COVID-19 pandemic on E-learning in Jordan is supplemented by [19]’s thorough evaluation of the acceptability of e-learning systems both before and during the pandemic. The significance of knowing what makes E-learning systems successful and popular is emphasized in both books. There is a good fit between this work and [19], which looks at what makes E-learning systems popular and successful, in terms of system quality, information quality, and user happiness. Supplementing this conversation, [20] and [21] investigate how student involvement and outcomes are affected by the usability of e-learning systems and teacher behavior. Findings from these research corroborate those from the article regarding the significance of high-quality systems and happy users. These research add to our knowledge of what makes E-learning systems effective, particularly in the context of a pandemic, by examining the interaction between technological usability and human factors, such as instructor conduct. [22–24] add more dimensions to understanding the long-term viability of e-learning platforms. With an eye toward the future, they survey emerging technology like the metaverse [24] and present models (such as the integrated ISSM and TAM model in [22] and the expanded D&M ISS model in [23]). Consistent with the paper’s emphasis on information and system quality as critical success elements in E-learning, these studies highlight the importance of self-directed learning, technical innovation, and other quality aspects. All things considered, the research presented in these articles weaves a complex web that both backs up and expands upon the paper’s conclusions. Particularly in light of recent, exceptional crises like the COVID-19 epidemic, they offer a comprehensive yet in-depth analysis of the many factors to be considered while developing and deploying E-learning systems in university.

This study aims to explore the impact of the COVID-19 pandemic on higher education in Jordan and the efficacy of E-learning portals. Recommendations to higher education decision-makers, including the Ministry of Higher Education and university stakeholders, will be provided to develop policies that ensure the success of E-learning portals. Conducted at Hashemite University in Jordan, this research aimed to identify the state of E-learning during the COVID-19 crisis, while adhering to the highest ethical standards and guidelines in the field. The primary critique of the study presented on its lack of novelty and limited academic contribution. While the manuscript meticulously details the impact of the COVID-19 pandemic on Jordanian higher education and the adaptation to E-learning, it largely reiterates findings and theoretical frameworks already established in existing literature. The reliance on the DeLone and McLean model, coupled with Shannon and Weaver’s information theory, provides a robust analytical foundation, but it does not introduce new theoretical perspectives or innovative methodologies. As such, the study predominantly reinforces pre-existing knowledge without substantially advancing the academic discourse on E-learning or offering fresh insights into the unique challenges and opportunities presented by the pandemic in the context of higher education. This limitation in originality and academic contribution could be addressed in future research by exploring uncharted territories, employing novel methodologies, or integrating interdisciplinary approaches to provide a more groundbreaking perspective on the subject.

The paper fills a significant need in the literature by investigating how the COVID-19 pandamic has affected Jordan’s higher education system, with an emphasis on the rise of online courses. It is reasonable to conduct this research in Jordan since the country’s educational system may face different problems and solutions in the wake of the pandemic than those in other parts of the world. The impacts of COVID-19 on education have been well documented in literature around the world, however this study stands out due to its targeted approach. Highlighting particular concerns such system quality, information quality, and user satisfaction, it offers insights into how educators and students in Jordan managed the transition to digital learning. For stakeholders in Jordan and comparable contexts to establish specific strategies for improving E-learning systems, these insights are crucial for lawmakers, educational institutions, and others. This study brings valuable insights into the educational issues caused by a pandemic in a particular country. It also helps in creating educational policies and practices that are more effective and tailored to the specific environment, which may be used to deal with future or current disruptions.

## 3. Materials and methods

This study employs a survey design to facilitate a quantitative analysis at Hashemite University, with the aim of assessing the current state of E-learning amidst the COVID-19 crisis and identifying the challenges that higher education in Jordan has faced during the pandemic. The research methodology is divided into two segments: descriptive and analytical. The descriptive segment delineates the characteristics of the sample and underscores the significance of each statement in the questionnaire. Concurrently, the analytical segment examines the relationship between variables and tests hypotheses using specific statistical techniques. The survey design is a valuable instrument that enables statistical analysis to assess the correlation and influence between variables [25]. This research leverages the “Statistical Package for Social Science” (SPSS) to analyze the mean and standard deviation for each statement related to the independent variables. Furthermore, it employs the smart partial least squares (PLS) method to test the hypotheses and the research model. The analysis utilizes exploratory factor analysis (EFA) and confirmatory factor analysis (CFA), as well as the measurement model (MM) and structural model (SM), to test the study’s hypotheses, as suggested by [26].

Data for this study were amassed via a survey using a questionnaire and were subsequently analyzed to assess the results and investigate the relationship between the independent components of the DeLone and McLean (D&M) model and the Shannon and Weaver information theory (S&W) contact levels, as noted by [27]. The S&W information theory comprises three accessible levels of contact, including the technical level (information system accuracy), semantic level, and effectiveness level (the influence of information on the receiver), as suggested by [28, 29]. The independent components of the D&M model consist of information quality, system quality, system usage, user satisfaction, service quality, and organizational effect, as detailed by DeLone and McLean [30].

### 3.1 Research model and hypothesis

This study aims to explore the interplay between the independent components of the DeLone and McLean (D&M) model and the contact levels of the Shannon and Weaver (S&W) information theory. In doing so, it utilizes system quality as a measure of technical success, and information quality to indicate semantic success. The effectiveness and success of these components are further assessed through metrics of user satisfaction, and their individual and organizational impact.

To thoroughly investigate this relationship, the study is guided by several research questions and hypotheses, which are rooted in the theoretical frameworks of the D&M model and S&W information theory. The questions are designed to dissect the influence of system quality, information quality, and service quality on system usage, user satisfaction, and the overall effectiveness of E-learning systems. They also explore the moderating roles of system usage and user satisfaction in these relationships.

The research questions that guide this study are:

1. Do system quality, information quality, and service quality have a significant effect on system usage?
2. Do system quality, information quality, and service quality have a significant effect on user satisfaction?
3. Do system quality and user satisfaction have a significant effect on the E-learning system?
4. Does system usage moderate the relationship between system quality, information quality, service quality, and E-learning?
5. Does user satisfaction moderate the relationship between system quality, information quality, service quality, and E-learning?

Based on these questions, the study formulates the following hypotheses:

- H0_1: System quality, information quality, and service quality significantly affect system use. This hypothesis is based on the premise that the technical and semantic aspects of a system influence its practical utilization.
- H0_2: System quality, information quality, and service quality significantly affect user satisfaction. The rationale here is that the overall quality of a system determines its acceptability and satisfaction among users.
- H0_3: System quality and user satisfaction significantly affect the E-learning system. This hypothesis stems from the belief that technical excellence and user contentment are key drivers of E-learning system effectiveness.
- H0_4: System use has a moderating role between system quality, information quality, service quality, and E-learning. This hypothesis is proposed under the assumption that the extent of system usage influences how quality factors affect E-learning outcomes.
- H0_5: User satisfaction has a moderating role between system quality, information quality, service quality, and E-learning. This is premised on the idea that user satisfaction can alter the impact of system qualities on the effectiveness of E-learning.

These hypotheses serve as the foundation for developing the conceptual framework of the research. They are integral in shaping the methodology and guiding the analysis of results, as will be elaborated in the subsequent sections.

### 3.2 Population and sample size

The population for this study encompasses students and staff engaged in E-learning in Jordan, with the sample being composed of Jordanian university students and employees from both the private and public sectors. The sample size for our study was meticulously determined using the methodology outlined by the research referenced in [31]. This approach ensured that the sample size met all the necessary criteria for the application of structural equation modeling (SEM). SEM is a complex statistical method used for testing and estimating causal relationships using an empirical approach. Additionally, the sample size adhered to the heuristic guidelines for the use of Smart PLS, a software tool designed for SEM, as suggested by the studies cited in [32, 33]. The research sample incorporated 392 participants. However, 26 responses were discarded due to the presence of extreme values. The decision to exclude certain data points was based on subjectivity, owing to inconsistencies found in the responses to different questions. For example, the responses to SysQ4 and INFQ3, as listed in Table 1, should exhibit consistency in their directional alignment. It is inconsistent to record disagreement for SysQ4 and agreement for INFQ3, suggesting that the respondent may not have thoroughly comprehended the questions, thereby warranting the exclusion of their response.

**Table 1:** The dimensions and their corresponding statements.

Quality System Corresponding Statements
**SysQ1: The student can easily upload the assignments and files to the E-learning system.**
**SysQ2: The personal information of the student on the E-learning system is preserved**, **and no one can see it.**
**SysQ3: The E-learning system suits the students’ educational needs.**
**SysQ4: The E-learning system is easy to use for students.**
**SysQ5: The E-learning system does not contain annoying ads.**
**Information Quality Corresponding statements**
**INFQ1: The E-learning system is organized so that the student can easily access it.**
**INFQ2: The information on the E-learning system is clear to students.**
**INFQ3: The information is consistent and useful for students.**
**INFQ4: The information on the E-learning system is available according to the student’s needs.**
**INFQ5: There is no information not related to the subject of study on the E-learning system.**
**Service Quality Corresponding statements**
**SERQ1: The service in the E-learning system is fast and useful.**
**SERQ2: Various services are available in the E-learning system that help students to learn.**
**SERQ3: The E-learning system allows for a teacher evaluation process by the student.**
**SERQ4: The E-learning system provides feedback to students directly from the professor.**
**SERQ5: The student can understand the professor through the E-learning system.**

A wide spectrum of Jordanian E-learning participants were studied, but university students and staff were particularly targeted because to their direct and intensive involvement with E-learning systems during the pandemic. Demarcation was necessary to gain relevant and focused insights on these systems’ use and perception. Both private and public university students and personnel were included to ensure a full grasp of E-learning from many perspectives in higher education. For an in-depth investigation of E-learning systems’ effectiveness, the sample was diverse to represent a wide range of experiences and perceptions.

The sample size was determined to ensure that it was representative and sufficient for sophisticated statistical analysis like SEM and Smart PLS. These methods demand a balance between statistical power and model complexity, making sample size crucial. After excluding 26 owing to discrepancies, the sample size of 392 offered a solid foundation for statistical analysis, assuring that the results were valid and applicable to Jordanian higher education. The removal of inconsistent responses guaranteed that the analysis was based on accurate and reliable data, reflecting a true understanding of the questionnaire items.

An extensive evaluation process determined which comments to delete due to extreme values. Each response was carefully examined for abnormalities and inconsistencies. This was done to verify that the data accurately represented respondents’ experiences and opinions. Since system quality (SysQ) and information quality (INFQ) are significant factors in assessing E-learning system efficacy, consistency in replies was crucial. The study sought to understand how the COVID-19 pandemic affected Jordanian higher education through E-learning by assuring data reliability and correctness.

### 3.3 Questionnaire design

The questionnaire is designed to cover the four following sections, the results of which are illustrated based on the common survey answers: strongly disagree, disagree, neutral, agree, and strongly agree:

1. The demographic section includes the respondent’s gender, age, education, current educational status, and college.
2. Independent dimensions with (27) items to represent three dimensions, quality system, information quality, and service quality. The corresponding statements for each independent dimension are listed in Table 1.
3. Moderating dimensions with (10) items to represent two dimensions: system use and user satisfaction. The corresponding statements for each independent dimension are listed in Table 2.
4. E-learning dimensions with (4) items to represent one dimension. The corresponding statements for each independent dimension are listed in Table 3.

**Table 2:** The moderating dimensions and corresponding statements.

System USE Corresponding Statements
**SYSUse1: Educational materials and lectures are shown on presentation programs such as PowerPoint.**
**SYSUse2: The E-learning system allows the sharing of pictures and files.**
**SYSUse3: The E-learning system allows video and audio calls.**
**SYSUse4: The E-learning system provides homework and learning practice examples.**
**SYSUse5: The E-learning system provides lessons and exams for materials.**
**User satisfaction Corresponding statements**
**UserSatis1: I have difficulty in dealing with the E-learning system.**
**UserSatis2: The E-learning system needs a strong internet.**
**UserSatis3: I make an effort to access the information I want within the E-learning system.**
**UserSatis4: I cannot show my personality in the E-learning system.**
**UserSatis5: There are services in the E-learning system that are difficult for me to use and benefit from.**

**Table 3:** The E-learning dimensions and corresponding statements.

The E-learning Corresponding Statements
**E-learning1: E-learning helped raise the level of students academically and raise the efficiency of educational achievement.**
**E-learning1: The use of E-learning tools provides me with many skills and information in a short time.**
**E-learning3: I consider the use of E-learning to be a method that keeps pace with modern trends in education.**
**E-learning4: I believe that the use of E-learning attracts students’ attention and provides learning opportunities for the largest number of them.**

### 3.4 Demographic sample characteristic

This study employs the Statistical Package for the Social Sciences (SPSS) to analyze the characteristics of the sample and determine the frequencies and percentages of the sample population. The demographic portion of the questionnaire is composed of five sections, which include questions on gender, age, education level, current educational status, and college. In terms of age, the ranges were predetermined prior to conducting the study, with the minimum age expected for incoming students set to less than 18. The subsequent age ranges are based on the typical age range of undergraduate and postgraduate students. Table 4 presents the frequencies and percentages of the responses to these questions in sequential order.

**Table 4:** The frequencies and percentage of the respondent’s answers to the questionnaire questions.

	Question	Frequency	Percent
**Gender**	Male	100	26.7
	Female	274	73.3
**Age**	<18	274	73.3
	19–25	59	15.8
	26–30	199	53.2
	>30	70	18.7
**Education level**	Bachelor’s degree	244	65.2
	Master’s degree	130	34.8
**Current educational Status**	Graduate	89	23.8
	To study	275	73.5
	Other	10	2.7
**Collage**	Sciences	53	14.2
	Nursing	33	8.8
	Engineering	88	23.5
	Medicine	9	2.4
	Pharmacy	6	1.6
	Economics and sciences	48	12.8
	Human resources	16	4.3
	Childhood	9	2.4
	Other	112	29.9

This study employs the Statistical Package for the Social Sciences (SPSS) to analyze the characteristics of the sample and determine the frequencies and percentages of the sample population. The demographic portion of the questionnaire is composed of five sections, which include questions on gender, age, education level, current educational status, and college. In terms of age, the ranges were predetermined prior to conducting the study, with the minimum age expected for incoming students set to less than 18. The subsequent age ranges are based on the typical age range of undergraduate and postgraduate students. Table 4 presents the frequencies and percentages of the responses to these questions in sequential order.

### 3.5 Face validity

To fulfill the objectives of this study, a questionnaire was crafted following a thorough review of previous research. This questionnaire was subsequently distributed to an expert panel to ensure the content’s meaningfulness, relevance, and clarity, and to establish face validity. This panel comprised five university experts who scrutinized the questionnaire for its face validity. Upon the collection of their feedback, necessary adjustments were made to the questionnaire in accordance with their comments and suggestions.

### 3.6 Ethics statement

The study is predicated on the participation of individuals who completed the survey. The authors obtained approval from their institution before proceeding. The research has been sanctioned by the Hashemite University Institutional Review Board (HU-IRB), with Agreement #2300463, dated 20/7/2023. Furthermore, participants were informed at the beginning of the survey that they were partaking in a study and completed the survey voluntarily, providing their consent. This project is started to be implemented on July 21, 2023, and concluded with the drafting of this paper around September 15, 2023.

### 3.6 Construct validity

Construct validity in measurement is intrinsically linked to the theoretical relationship of a variable with other variables. It pertains to the degree to which a measure aligns with the construct it is designed to gauge, in correlation with the established measures of other constructs. Cook and Campbell [15] characterize it as the extent to which a construct is measured utilizing the selected variables that encapsulate the concept of interest. In this study, the Kaiser–Meyer–Olkin (KMO) test is employed to evaluate the adequacy of sampling for the variables within the model and for the model as a whole. The KMO value should exceed 0.5, signifying satisfactory sampling. Moreover, given the sample size, the loading factor ought to surpass a threshold value of 0.3.

Factor analysis was utilized to ascertain the factor loadings of all the statements within the questionnaires. This includes the dimensions of the independent variable, moderating variable, and E-learning, thereby assessing construct validity. The consequent values delineated in Table 5 for the loading factors (LF) surpass the threshold value of 0.3, and the KMO values exceed 0.5 for all statements in the questionnaire. These values suggest that the questionnaire statements are suitable for analysis.

**Table 5:** Factor analysis loadings for independent variables.

Statement	LF	KMO	Statement	LF	KMO
**Quality System**			System Use		
**SysQ1**	0.764	SYSUse1	SYSUse1	0.745	0.838
**SysQ2**	0.705		SYSUse2	0.500	
**SysQ3**	0.741		SYSUse3	0.558	
**SysQ4**	0.820		SYSUse4	0.817	
**SysQ5**	0.795		SYSUse5	0.796	
**Information Quality**			User Satisfaction		
**INFQ1**	0.496	0.849	UserSatis1	0.666	0.547
**INFQ2**	0.487		UserSatis2	0.680	
**INFQ3**	0.654		UserSatis3	0.513	
**INFQ4**	0.778		UserSatis4	0.758	
**INFQ5**	0.760		UserSatis5	0.685	
**Service Quality**			E-Learning		
**SERQ1**	0.742	0.869	E-learning1	0.918	0.558
**SERQ2**	0.771		E-learning2	0.522	
**SERQ3**	0.614		E-learning3	0.758	
**SERQ4**	0.744		E-learning4	0.795	
**SERQ5**	0.760				

### 3.7. Reliability test

Reliability pertains to the extent to which measures are devoid of error and consistently produce the same results, thereby signifying the reliability of a procedure. If a measuring device or method consistently delivers the same score for individuals or statements of equivalent values, it is deemed reliable. To evaluate the reliability of the questionnaire, Cronbach’s α level was utilized as a criterion of internal consistency. The values of Cronbach’s alpha (α) ought to exceed 0.60, signifying internal consistency amongst the questionnaire statements [34].

Calculations were conducted for the dimensions of the independent variables, and the resultant values of Cronbach’s alpha are showcased in Table 6. For the dimensions of independent variables, moderating variables, and the dependent variable represented by E-learning, all values exceed 0.60, indicating internal consistency amongst the questionnaire statements.

**Table 6:** Cronbach’s alpha for independent variables, moderating variables, and the dependent variables.

Dimension	α Value
**Independent Variables**	
**Quality system**	0.823
**Information quality**	0.853
**Service quality**	0.904
**All dependent variables**	0.825
**Moderator Variables**	
**System use**	0.884
**User satisfaction**	0.821
**All moderator variables**	0.787
**Dependent Variables**	
**E-learning**	0.825

### 3.8 Collinearity test

Collinearity refers to a scenario in which a number of independent variables exhibit high correlation, which could potentially inflate the variance of at least one estimated regression coefficient. To confirm that there is no issue of collinearity in the correlation between the independent variables, we calculated the variance inflation factor (VIF), as shown in Table 7. VIF is a statistical measure that quantifies the intensity of multicollinearity in an ordinary least squares regression analysis. It demonstrates how much the variance of an estimated regression coefficient is increased due to collinearity. According to Talwar et al. [35], the value of VIF, which equals 1 divided by the tolerance, must be less than 10 (implying tolerance must exceed 0.1) to ensure there is no collinearity issue with these variables. We calculated the VIF factor values for all dimensions of the independent variables and moderating variables, as shown in Table 8. All values are less than 10 with a tolerance (T) greater than 0.1, indicating that there is no issue of collinearity with these variables. Because the dependent variable comprises only one variable, there is no collinearity problem.

**Table 7:** VIF of dimensions for independent variables and moderating variables.

Dimension	*T*	*VIF*
**Independent Variables**		
**Quality system**	0.362	2.762
**Information quality**	0.360	2.778
**Service quality**	0.984	1.016
**Moderator Variables**		
**System use**	0.718	1.393
**User satisfaction**	0.718	1.393

**Table 8:** Indicators of perfect fit to model of study.

	RMSEA
**Independent variable**	0.056
**Moderating variable**	0.069
**E-learning dimensional**	0.00
**Model of study**	0.056

### 3.9 Confirmatory factor analysis for the variables and the model of study

**Fig 1:**
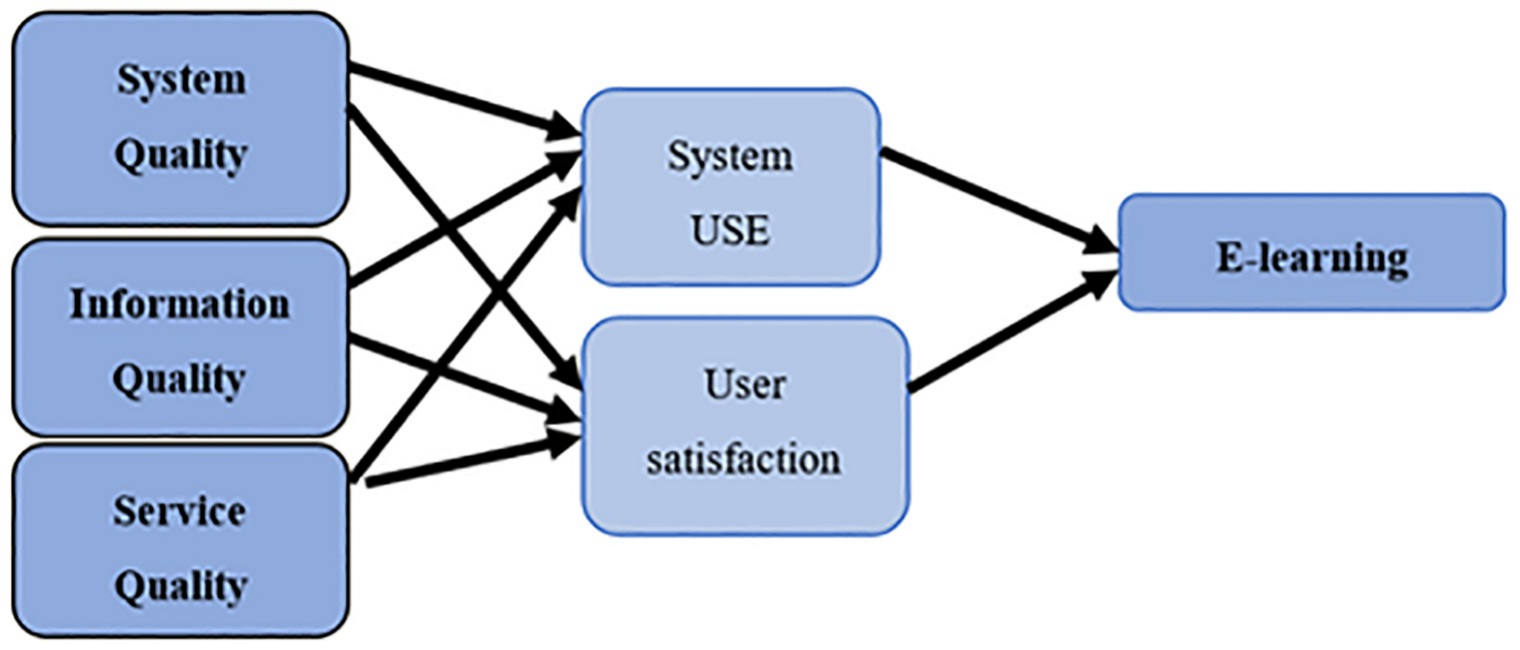
The research model.

The statistical analysis results, derived from the responses of the individuals in the study sample concerning the study variables and their dimensions, have been described utilizing the specific statistical means delineated in the research methodology. This includes the arithmetic mean and standard deviation for all study variables, followed by the testing of the study hypotheses using the EMOS software. To calculate the comparative alignment indicators’ values, we employed the root mean square error of approximation (RMSEA). The confirmatory factor analysis for the variables and the study model, presented in Table 8, indicates that the RMSEA value is close to zero, signifying an excellent fit, high quality of compliance, and the validity of the statements in the study model scale. Fig 1 provides a visual representation of the confirmatory analysis of the study model with its statements.’

## 4. Results

The results are discussed in detail in this section. The analysis is divided into statistical analysis, including hypothesis testing, and path analysis.

### 4.1 Confirmatory factor

The “Statistical Package for Social Science” (SPSS) is a Windows-based program used for statistical analysis, including data entry, analysis, and for the creation of tables and graphs. For this study, SPSS was used to analyze the mean (m), standard deviation (σ), and rank (R) for each statement of the independent variables. Table 9 displays the results for the dimensions of all variables in this study, which are in the mid-evaluation score level, except for the service quality statements which have a high evaluation score.

**Table 9:** The results of statistical analysis for each statement.

Statement	*m*	σ	*R*	Level	Statement	*m*	σ	*R*	Level
**Quality System**					System Use				
**SysQ1**	3.55	1.35	2	mid	SYSUse1	3.38	1.16	5	mid
**SysQ2**	3.26	1.42	5	mid	SYSUse2	3.42	0.97	4	mid
**SysQ3**	3.28	1.40	4	mid	SYSUse3	4.07	0.96	1	high
**SysQ4**	3.52	1.36	3	mid	SYSUse4	3.65	1.23	3	mid
**SysQ5**	3.59	1.43	1	mid	SYSUse5	3.67	1.19	2	mid
**Overall**	3.44	1.06	-	mid	Overall	3.64	0.92	-	mid
**Information Quality**					User Satisfaction				
**INFQ1**	3.45	1.24	2	mid	UserSatis1	3.76	1.16	1	high
**INFQ2**	3.36	1.12	4	mid	UserSatis2	3.29	1.39	4	mid
**INFQ3**	3.60	1.36	1	mid	UserSatis3	3.39	1.26	3	mid
**INFQ4**	3.33	1.23	5	mid	UserSatis4	3.06	1.31	5	mid
**INFQ5**	3.39	1.23	3	mid	UserSatis5	3.70	0.98	2	high
**Overall**	3.43	0.98	-	mid	Overall	3.44	0.64	-	mid
**Service Quality**					E-Learning				
**SERQ1**	3.74	1.30	2	high	E-learning1	3.62	1.07	1	mid
**SERQ2**	3.82	1.24	2	high	E-learning2	3.19	1.10	3	mid
**SERQ3**	3.59	1.35	5	mid	E-learning3	2.84	1.24	4	mid
**SERQ4**	3.70	1.26	4	high	E-learning4	3.60	1.15	2	mid
**SERQ5**	3.71	1.31	3	high	Overall	3.31	0.64	-	mid
**Overall**	3.71	1.10	-	high					

### 4.2 Hypothesis testing

Based on the study model, five primary null hypotheses were formulated and analyzed using the AMOS program. The objective was to investigate the relationship and effect of the independent variables on the dependent variable and determine whether the established hypotheses were accepted or rejected.

The following hypotheses were analyzed:

H0_1: System quality, information quality, and service quality significantly affect the system use.
H0_2: System quality, information quality, and service quality significantly affect the user satisfaction.
H0_3: User satisfaction and user satisfaction significantly affect the E-learning.
H0_4: System use has a moderating role between system quality, information quality, service quality, and E-learning.
H0_5: User satisfaction has a moderating role between system quality, information quality, service quality, and E-learning.

The aim of this study was to elucidate the relationship between the dimensions of system quality, information quality, and service quality, and their impacts on the independent variables, moderating variables, and E-learning. The outcomes obtained from the AMOS software provide data regarding the determination coefficient of the correlation (R2), the slope parameter within the regression model (β), the computed t-values, and the significance level (*p*-value < 0.05). Furthermore, the F-test was performed to examine the equality of variances utilizing hypothesis testing. If the result of the F-test, as shown by the F value in Tables 10–12, is statistically significant, the null hypothesis can be dismissed. Conversely, if the result is not statistically significant, the null hypothesis cannot be discarded [36]. Moreover, the T-test was conducted in this study using the sample derived from this research, thus enabling a comparison between the study sample and the null hypothesis. The outcome of this test is denoted by the T value in Tables 10–12 [37].

**Table 10:** Effect of independent variable on system use.

Independent Variable	β	T	*p*-Value
Quality system	−0.079	−2.005	0.046
Information quality	0.086	2.154	0.032
Service Quality	0.890	37.069	0.000
	R^2^	0.790	
	F	464.243	0.00
	Β	0.851	

**Table 11:** Effect of independent variable on user satisfaction.

Independent Variable	β	T	Sig
Quality system	0.041	0.565	0.572
Information quality	0.159	2.197	0.029
Service Quality	0.541	12.366	0.000
	R^2^	0.304	
	F	53.743	0.00
	B	1.826	

**Table 12:** Significant effect of system quality and user satisfaction on E-learning.

Independent Variable	β	T	Sig
Service Quality	−0.053	−1.057	0.291
User satisfaction	0.280	5.574	0.000
	R^2^	0.078	
	F	15.628	0.00

Based on these values, the posited hypotheses are either accepted or rejected. Pertaining to the first hypothesis (H0_1), the test results as illustrated in Table 10 reveal a potent and positive influence of the independent variables on system use. Consequently, the null hypothesis is discarded, and the alternative hypothesis is accepted.

Concerning the second hypothesis (H0_2), the results from the test in Table 11 indicate a positive and strong effect of the independent variables on user satisfaction. Therefore, the null hypothesis is rejected, and the alternative hypothesis is accepted.

In regard to the third hypothesis (H0_3), the results of the test in Table 12 demonstrate a positive and strong effect of system quality and user satisfaction on E-learning. Hence, the null hypothesis is rejected, and the alternative hypothesis is accepted.

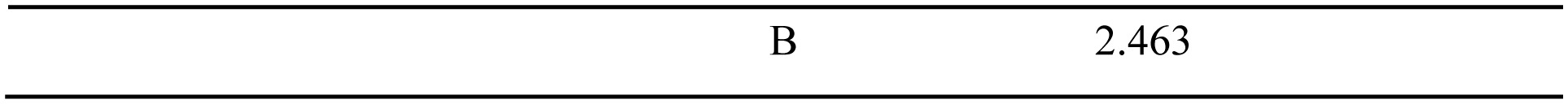


**Fig 2:**
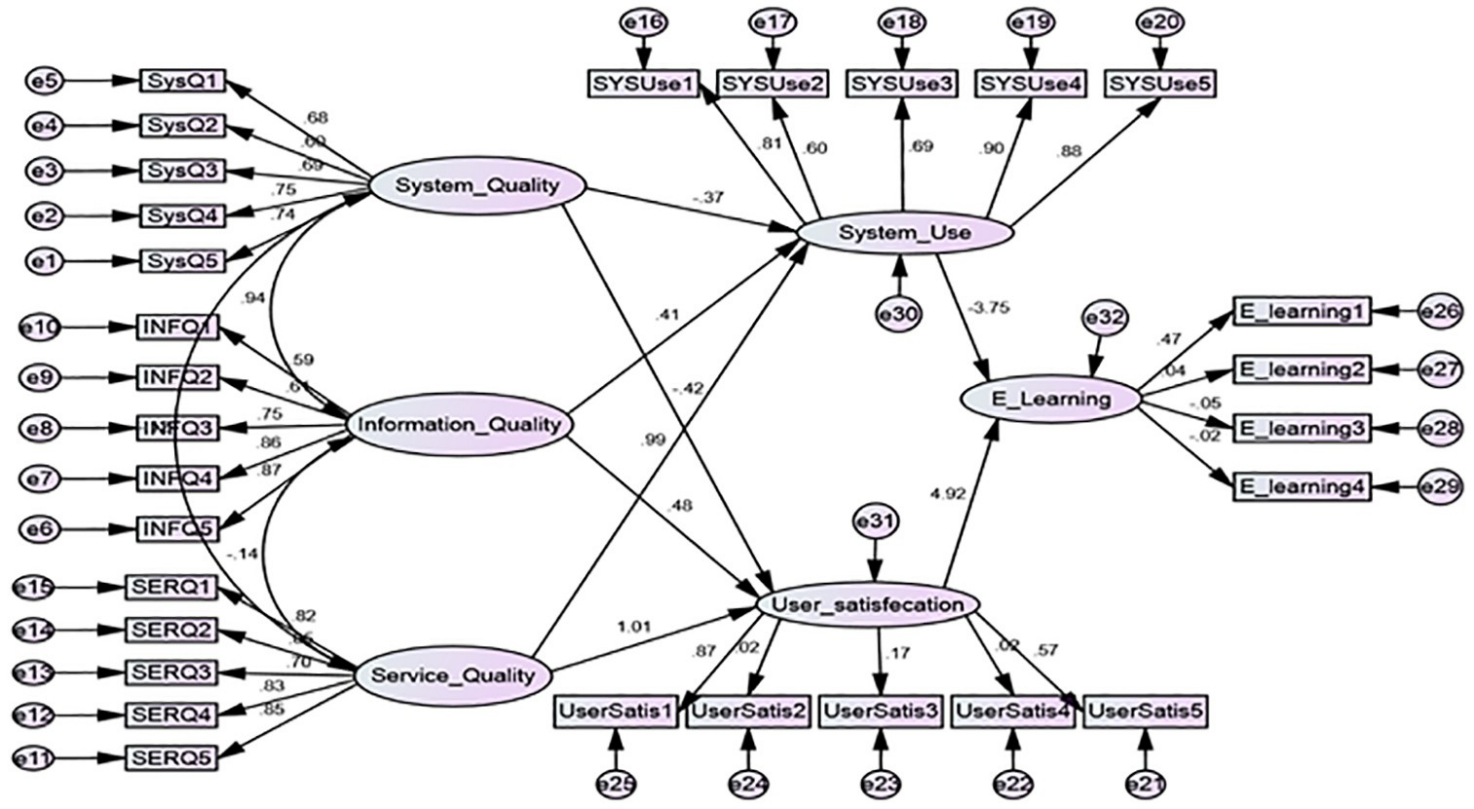
Confirmatory factor analysis for model of study.

Regarding the fourth hypothesis (H0_4), the structure model depicted in Fig 2 suggests that system use plays a moderating role between system quality, information quality, service quality, and E-learning.

**Fig 3:**
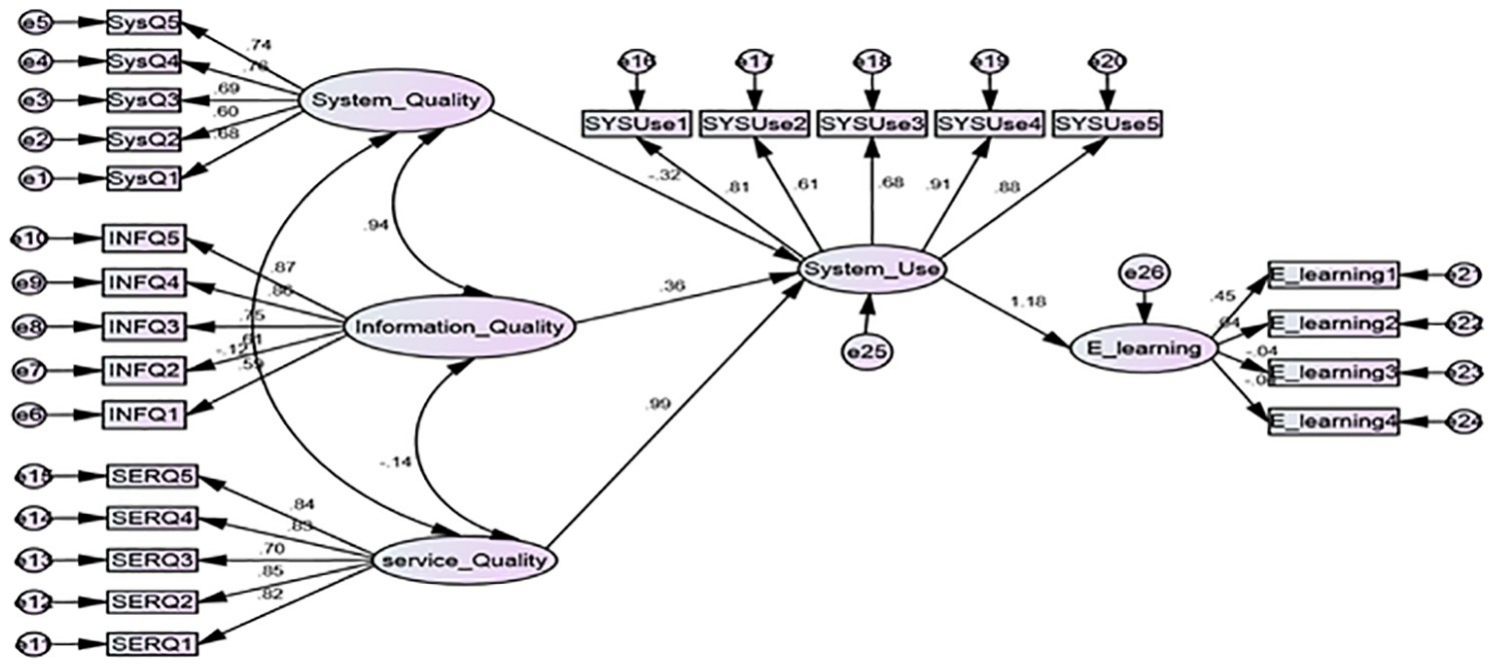
The moderating role between system quality, information quality, service quality, and E-learning.

Based on the structural model presented in Fig 3, the fifth hypothesis (H0_5) indicates that user satisfaction plays a moderating role between system quality, information quality, service quality, and E-learning.

### 4.3 Path analysis system

The path analysis method was employed to study and analyze the relationships between one or more independent variables, irrespective of whether these variables are continuous or intermittent. A significance level of 0.05 was adopted to determine the significance of the effect. The computed level of significance was compared with the approved level of significance, and the effects were considered statistically significant if they were indeed significant. The researcher analyzed the second hypothesis of the study (H0_1), which postulates that “system Use has a moderating role between system quality, information quality, service quality, and E-learning,” by ascertaining the value of the direct effect of system quality, information quality, and service quality on user satisfaction. The results, as displayed in Table 13, point to a potent and positive impact of the independent variables (system quality, information quality, and service quality) on system use, as well as a statistically significant direct effect of system quality on system use.

**Table 13:** Path analysis for the first hypothesis of the study (H0_1).

			Estimate	S.E.	C.R.	*p*
System Use	<---	System Quality	−0.329	0.166	−1.983	0.047
System Use	<---	Information Quality	0.46	0.208	2.212	0.027
System Use	<---	Service Quality	0.884	0.049	17.856	
E-Learning	<---	System Use	0.598	0.056	10.695	

To address this hypothesis, the researcher analyzed the third hypothesis of the study (H0_3), which posits “system quality and user satisfaction have a moderating role between E-learning and independent variables”. This was achieved by determining the value of the direct effect of system quality, information quality, and service quality on E-learning. The results, displayed in Table 14, suggest a positive and robust influence of the independent variables (system quality, information quality, and service quality) on system use, as well as a positive and potent impact of the independent variables (system quality, information quality, and service quality) on user satisfaction. Additionally, there is a statistically significant direct effect of both system quality and user satisfaction on E-learning.

**Table 14:** Path analysis for the first hypothesis of the study (H0_2).

			Estimate	S.E.	C.R.	*p*
User Satisfaction	<---	System Quality	−0.486	0.232	−2.096	0.036
User Satisfaction	<---	Information Quality	0.723	0.291	2.488	0.013
User Satisfaction	<---	Service Quality	0.951	0.048	19.817	
E-Learning	<---	User Satisfaction	0.597	0.051	11.735	

## 5. Discussion

The findings from this study contribute to the understanding of the pivotal role that system quality, information quality, and service quality play in influencing system use and user satisfaction. The high scores for service quality suggest that users place significant importance on this aspect when interacting with the system.

The rejection of H0_1 indicates that better system quality, information quality, and service quality are likely to enhance system use. This is crucial for developers and service providers who aim to increase user engagement with their systems. The rejection of H0_2 suggests that these quality factors are also critical for user satisfaction, which can lead to improved user retention and positive word-of-mouth for the service or product. The results supporting the dismissal of H0_3 emphasize the importance of system quality and user satisfaction in promoting E-learning. This underlines the fact that E-learning platforms must not only be well-designed but must also meet the users’ satisfaction to be effective.

The structural models depicted in Figs 2 and 3 and the results from Tables 13 and 14 suggest that both system use and user satisfaction are not merely outcomes but are also pivotal in enhancing the effectiveness of E-learning. Their role as moderators indicates that they can influence the extent to which the system’s quality aspects impact E-learning outcomes. The study expands on existing models by demonstrating that the relationship between system quality factors and E-learning is not linear but is moderated by system use and user satisfaction. This nuanced understanding can help in the design of more effective educational systems and services.

Global Higher Education Systems and E-learning Adaptation: This report shows how the COVID-19 epidemic has changed education worldwide, including Jordan’s higher education system’s move to E-learning. This change affects universities globally, not just Jordan. Rapid acceptance of digital learning and teaching platforms has shown the potential for a more flexible, accessible, and varied educational environment. However, it emphasizes the necessity for strong digital infrastructures and good online learning pedagogy. E-learning is a global trend, thus higher education systems must rethink their teaching methods, curricula, and evaluation methods to suit remote and hybrid learning. Challenges and Opportunities for International Collaboration: The findings also emphasize the obstacles institutions confront in adjusting to online education, such as digital divide, faculty and student digital literacy, and technical and pedagogical support. International higher education partnership is possible with these challenges. Sharing E-learning resources, experiences, and best practices benefits universities worldwide. Joint online courses, multinational webinars, and shared digital materials can improve online education and make it more inclusive and egalitarian. Collaborations could enable creative pandemic solutions and a more connected and resilient global educational community. Future Policy and Practice Directions: This research has major consequences for higher education policy. Policymakers must examine both infrastructure and pedagogical changes to enable E-learning. This entails training teachers, providing equal technological access, and creating regulations that balance online and in-person learning. To suit students’ and instructors’ changing demands, E-learning solutions must be evaluated and researched often. Higher education systems can learn from the pandemic and create more robust and flexible policies and procedures to tackle future health emergencies, environmental issues, and technological advances.

This study has several practical consequences for Jordanian higher education authorities, educators, and technology developers. The study emphasizes the importance of system, information, and service quality in E-learning platform effectiveness. This implies investing heavily in improving these features, such as upgrading the user interface, system dependability, and support services. User happiness is also crucial to E-learning uptake and success, according to the study. Universities and E-learning providers should include regular feedback methods to adjust and develop their systems to user needs.

The report also emphasizes the need for digital literacy training for instructors and students to effectively use E-learning platforms. These programs could reduce the digital divide and ensure great education for all. This research could also help create strategies to make E-learning a permanent part of education rather than a crisis response. Develop blended learning approaches that combine the best of traditional and online education.

This paper analyzes the COVID-19 pandemic’s impact on higher education through E-learning in a specific regional setting, adding to current knowledge. The study’s focus on Jordan provides unique insights into the problems and prospects of adopting E-learning in crises worldwide. It applies the DeLone and McLean model and Shannon and Weaver’s information theory to a pandemic, providing a new viewpoint on these theories.

This study also expands E-learning studies. Longitudinal studies could examine how the quick shift to E-learning has changed teaching and learning methods. Comparative studies of E-learning’s efficiency in different cultures and socioeconomic circumstances are possible. E-learning’s psychological effects on students and educators, including motivation, engagement, and mental health, should be studied. Finally, this study encourages multidisciplinary research on technology and education, which could lead to breakthrough E-learning solutions.

## 6. Conclusion

The study presented in Manuscript 100 offers a nuanced analysis of the impact of the COVID-19 pandemic on Jordan’s higher education system, with a specific focus on the adaptation to E-learning. From an academic standpoint, this research contributes significantly to the existing body of knowledge by contextualizing the DeLone and McLean model and Shannon and Weaver’s information theory within the unique framework of Jordan’s higher education during a global crisis. This contextualization not only validates these models in a new setting but also reveals specific nuances and implications that are pertinent to similar educational systems in other regions. The practical implications of this study are manifold. Firstly, it underscores the critical role of system quality, information quality, and service quality in enhancing user satisfaction and system usage in E-learning platforms. This insight is invaluable for educational policymakers and administrators in Jordan and comparable contexts, as it provides a clear direction for where resources and efforts should be concentrated to improve online education. The study also makes a strategic suggestion that educational institutions might implement: creating a dedicated department to help students adjust to E-learning platforms. Not only is it necessary to constantly modify and enhance E-learning platforms in response to urgent emergencies like the COVID-19 epidemic, but this should be done both in the short and long term as an educational strategy, according to the study. The study’s findings in this area suggest that blended learning models may soon be the standard, bringing both students and teachers closer to a world where technology plays an integral role in the classroom. In conclusion, while this study reaffirms some known aspects of E-learning, its real contribution lies in its detailed analysis of these aspects within the specific context of the Jordanian higher education system. This provides a template for other nations with similar educational and socio-economic dynamics to draw lessons and insights. Future research could build on this study’s findings by exploring longitudinal impacts of E-learning post-pandemic and its role in shaping new norms in higher education globally.

## 7. Limitation of the research

The manuscript analyses how the COVID-19 epidemic affected Jordan’s higher education system, specifically E-learning adoption. However, study limitations must be acknowledged. The research mostly focuses on Hashemite University, which may not fully represent the different experiences and issues of other Jordanian universities. The findings may not apply to all Jordanian higher education institutions due to this constraint. Second, the study focuses mainly on quantitative survey data, which may not convey the depth and variety of human experiences and viewpoints on E-learning transition. Interviews or focus groups may reveal more about students and faculty’s complex issues and opportunities. The quick evolution of the pandemic and E-learning technologies indicate that the study’s findings may not apply to future crises or other crises. Future research could overcome these limitations by include more institutions, using mixed-method approaches, and studying the pandemic’s long-term effects on higher education.

Further research might examine the long-term effects of the COVID-19 pandemic on higher education worldwide, focusing on varied educational contexts and crisis management strategies. To understand the wider effects of E-learning and digital transformation in education, comparative studies across institutions and nations are needed. Qualitative research approaches include in-depth interviews and case studies can also reveal student, faculty, and administration perspectives. Also useful would be studying blended learning methods’ post-pandemic efficacy and ability to change educational practices and policies. Artificial intelligence and virtual reality can improve E-learning experiences, which can illuminate the future of digital education. Finally, psychological and socio-economic studies on students and educators can provide a more complete picture of the pandemic’s effects on schooling.

## Supporting information

S1 Data The initial data from the survey is available in the attached DOCX file. (DOCX)
