# Supplementary material for: The effect of the COVID-19 pandemic crisis on the Jordanian higher education system
Source: PLoS One. 2024 Apr 19;19(4):e0299531. doi: 10.1371/journal.pone.0299531 (PMC11029628; doi:10.1371/journal.pone.0299531)
Supplement: S1 Data — (DOCX) [file pone.0299531.s001.docx]

**Raw Data**

A total of 374 participants took part in the survey. Their information is summarized in Table 1, while the raw data from the survey are summarized in Table 2.

**Table 1.** The frequencies and percentage of the respondent’s answers to the questionnaire questions.

|  | Question | Frequency | Percent |
| --- | --- | --- | --- |
| **Gender** | Male | 100 | 26.7 |
|  | Female | 274 | 73.3 |
| **Age** | <18 | 274 | 73.3 |
|  | 19–25 | 59 | 15.8 |
|  | 26–30 | 199 | 53.2 |
|  | >30 | 70 | 18.7 |
| **Education level** | Bachelor’s degree | 244 | 65.2 |
|  | Master’s degree | 130 | 34.8 |
| **Current educational Status** | Graduate | 89 | 23.8 |
|  | To study | 275 | 73.5 |
|  | Other | 10 | 2.7 |
| **Collage** | Sciences | 53 | 14.2 |
|  | Nursing | 33 | 8.8 |
|  | Engineering | 88 | 23.5 |
|  | Medicine | 9 | 2.4 |
|  | Pharmacy | 6 | 1.6 |
|  | Economics and sciences | 48 | 12.8 |
|  | Human resources | 16 | 4.3 |
|  | Childhood | 9 | 2.4 |
|  | Other | 112 | 29.9 |

**Table 3.2:** Research Questionnaire

| **Paragraph** | | | | | | | |
| --- | --- | --- | --- | --- | --- | --- | --- |
| 1. **System Quality:** A set of procedures and processes that focus on achieving the objectives of the system to meet the needs of Users according to the required conditions | | | | | | | |
| **No** | **Statement** | **Strongly Disagree** | **Disagree** | **Neutral** | **Agree** | **Strongly Agree** |  |
|  | The student can easily upload the assignments and files to the e-learning system | 10 | 88 | 50 | 138 | 88 |  |
|  | The personal information of the student on the e-learning system is preserved and no one can see it. | 21 | 83 | 131 | 56 | 83 |  |
|  | The e-learning system suits the students ’educational needs | 25 | 75 | 119 | 80 | 75 |  |
|  | The e-learning system is easy to Use for students | 15 | 81 | 54 | 143 | 81 |  |
|  | The e-learning system does not contain annoying ads | 15 | 89 | 19 | 162 | 89 |  |
| 1. **Information Quality:** It is a set of tools that may be in the form of a program, aiming to increase the emphasis on the final outputs of the information system. | | | | | | | |
| **No** | **Statement** | **Strongly Disagree** | **Disagree** | **Neutral** | **Agree** | **Strongly Agree** |  |
|  | The e-learning system is organized so that the student can easily access it | 12 | 75 | 95 | 117 | 75 |  |
|  | The information on the e-learning system is clear to students | 13 | 64 | 138 | 95 | 64 |  |
|  | The information is consistent and Useful for students | 9 | 91 | 32 | 151 | 91 |  |
|  | The information on the e-learning system is available according to the student’s needs | 11 | 78 | 140 | 67 | 78 |  |
|  | There is no information not related to the subject of study on the e-learning system | 13 | 73 | 116 | 99 | 73 |  |
| 1. **Quality of Service:** It is the ability to provide different priority to different applications, Users, or data flows, or to ensure a certain level of performance for the data flow. | | | | | | | |
| **No** | **Statement** | **Strongly Disagree** | **Disagree** | **Neutral** | **Agree** | **Strongly Agree** |  |
|  | The service in the e-learning system is fast and Useful | 5 | 69 | 65 | 111 | 124 |  |
|  | Various services are available in the e-learning system that help students to learn | 1 | 70 | 55 | 117 | 131 |  |
|  | The e-learning system allows the teacher evaluation process by the student | 6 | 72 | 108 | 71 | 117 |  |
|  | The e-learning system provides feedback to students directly from the professor | 6 | 66 | 72 | 118 | 112 |  |
|  | The student can understand the professor through the e-learning system | 12 | 62 | 57 | 134 | 109 |  |
| 1. **Use of the system** | | | | | | | |
| **No** | **Statement** | **Strongly Disagree** | **Disagree** | **Neutral** | **Agree** | **Strongly Agree** |  |
|  | Educational materials and lectures are shown on presentation programs such as PowerPoint | 10 | 68 | 140 | 82 | 74 |  |
|  | The e-learning system allows sharing pictures and files | 14 | 46 | 131 | 135 | 48 |  |
|  | The e-learning system allows video and audio calls | 12 | 14 | 54 | 150 | 144 |  |
|  | The e-learning system provides homework and learning practice examples | 9 | 64 | 72 | 133 | 96 |  |
|  | The e-learning system provides lessons and exams for materials | 15 | 56 | 47 | 175 | 81 |  |
| 1. **User satisfaction:** meeting the needs and requirements of customers, responding to all their inquiries, and securing their requirements on time, according to the required characteristics, and within the specified conditions, in a manner that ensures their continued interaction with them. | | | | | | | |
| **No** | **Statement** | **Strongly Disagree** | **Disagree** | **neutral** | **Agree** | **Strongly Agree** |  |
|  | I feel difficult in dealing with the e-learning system | 6 | 70 | 26 | 178 | 94 |  |
|  | The e-learning system needs a strong internet | 16 | 86 | 133 | 51 | 88 |  |
|  | I make an effort to access the information I want within the e-learning system | 10 | 81 | 117 | 85 | 81 |  |
|  | I cannot show my personality in the e-learning system | 42 | 52 | 179 | 44 | 57 |  |
|  | There are services in the e-learning system that are difficult for me to Use and benefit from | 8 | 43 | 79 | 167 | 77 |  |
| 1. **E-learning** | | | | | | | |
| **No** | **Statement** | **Strongly Disagree** | **Disagree** | **neutral** | **Agree** | **Strongly Agree** |  |
|  | E-learning helped raise the level of students academically and raise the efficiency of educational achievement | 16 | 38 | 89 | 160 | 71 |  |
|  | The use of e-learning tools provides me with many skills and information in a short time | 9 | 75 | 201 | 14 | 75 |  |
|  | He considered that the use of e-learning is a method that keeps pace with modern trends in education | 38 | 134 | 128 | 0 | 74 |  |
|  | I believe that the use of e-learning attracts students' attention and provides learning opportunities for the largest number of them | 5 | 61 | 107 | 107 | 94 |  |
